# Association of dose escalation of octreotide long-acting release on clinical symptoms and tumor markers and response among patients with neuroendocrine tumors

**DOI:** 10.1002/cam4.435

**Published:** 2015-02-26

**Authors:** Khalid Al-Efraij, Mohammed A Aljama, Hagen Fritz Kennecke

**Affiliations:** 1 Division of Respirology, University of British ColumbiaVancouver, British Columbia, Canada; 2 Division of Hematology, University of British ColumbiaVancouver, British Columbia, Canada; 3 Division of Medical Oncology, British Columbia Cancer Agency, University of British ColumbiaVancouver, British Columbia, Canada

**Keywords:** 5-Hydoxyindoacetate, chromogranin A, clinical symptoms, intramuscular, NET, neuroendocrine tumors, O-LAR

## Abstract

Patients with nonresectable metastatic neuroendocrine tumors (NETs) experience symptoms of hormone hypersecretion including diarrhea, flushing, and bronchoconstriction, which can interfere with quality of life [Anthony and Vinik (2011) *Pancreas*, 40:987]. Treatment with a long-acting release formulation of octreotide, a somatostatin analog, can help to alleviate these symptoms. Although high doses of octreotide are often required for adequate symptom control, the relationship between octreotide dose escalation and symptom control in the NET context is not well quantified in the literature. A retrospective chart review was conducted of nonresectable metastatic NET patients who received a dose greater than 30 mg intramuscular octreotide long-acting formulation (O-LAR) at any time between January 2005 and December 2011 at the British Columbia Cancer Agency (BCCA). The association between dose escalation of O-LAR, chromogranin A (CGA), 24-h urine 5-hydoxyindoacetate (5-HIAA), symptom control, and radiological progression was explored. Dose escalation of O-LAR was associated with improved symptom control in NET patients who were refractory to the standard dose levels. Reduction of serum CGA & 5-HIAA levels by at least 10% was observed in 31% and 23% respectively. Retrospective review of imaging did not document any reductions in tumor volume. Higher doses of O-LAR are associated with improved symptom control in NET patients. The variability in tumor marker levels in response to O-LAR dose escalation may indicate that tumor marker levels may not be an accurate assessment of therapeutic efficacy.

## Introduction

Neuroendocrine tumors (NETs) are uncommon malignancies with an incidence of 0.9–1.3 per 100,000 persons per year 1–3. The tumors are derived from endodermal cells with a secretory capacity and can originate in the fore-, mid-, and hindgut. Patients with NET metastases are symptomatic from hypersecretion of vasoactive amines and peptides rather than from tumor bulk. The symptoms of hormone hypersecretion from symptomatic secretory NET include diarrhea, flushing, and bronchoconstriction 4.

Most NETs contain a high density of somatostatin receptors 5. Receptor subtypes 2 and 5 are the most important for symptom control, and are specifically targeted by somatostatin analogs octreotide and lanreotide, respectively. Somatostatin analogs have proven to be efficacious in controlling excessive hormonal secretions in NET patients 6,7, with 30–70% of treated patients demonstrating a stabilization of secretory symptoms lasting from months to a few years 8–11. Long-term studies have shown somatostatin analogs to be safe, with the most significant adverse event being development of gallstones.

Octreotide long-acting release (O-LAR) is indicated for long-term treatment of the diarrhea and flushing episodes associated with symptomatic secretory NET. The dose of O-LAR approved by the Food and Drug Administration is 20–30 mg administered intramuscularly on a monthly basis 12. However, in this population, O-LAR dose escalation is common and 20–40% of NET patients receive higher concentrations to control symptoms 13 and this is well tolerated 14. Although the impact of O-LAR on disease control is not well described in the literature, some have reported antiproliferative effects and radiologic responses in up to 30% of cases with high-dose somatostatin analogs 11,15,16.

We conducted a retrospective analysis to fully understand if higher doses of O-LAR might successfully mitigate symptoms in NET patients that remain symptomatic in spite of standard doses of LAR (30 mg intramuscular [IM] q month), by evaluating the association between dose escalation of O-LAR, symptom control, tumor markers (chromogranin A [CGA] and 5-hydoxyindoacetate [5-HIAA]), and radiological progression of the tumors.

## Patients and Methods

This study included NET patients who were referred to one of the five British Columbia (BC) Cancer Agency clinics across the Province. BC Cancer Agency is the single payer for all cancer therapeutics in BC, a Canadian province with 4.4 million inhabitants. This study was conducted with approval of the University of British Columbia—British Columbia Cancer Agency (BCCA) research ethics board.

A list of all BC Cancer Agency patients who received O-LAR between January 2005 and December 2011 was provided by BCCA pharmacy. A total of 265 unique patients received a dose of O-LAR during the study period. Of this group, 37 (14%) patients received an escalated dose, defined as any initial or subsequent increase in dose of O-LAR above 30 mg IM q month. This group represented the study cohort. A full chart review was conducted of relevant clinical, imaging, and laboratory parameters and data were analyzed until April 2013.

The baseline characteristics of study cohort including age, sex, tumor origin, prior treatment, and the site of metastasis at the time of dose escalation, the dose, and the reason for dose escalation were documented.

Symptom frequency was compared pre- and post dose escalation. Symptoms including diarrhea and flushing were reported according to Common Terminology Criteria for Adverse Events (CTCAE) version 4.0 17. A reduction in symptoms from Grades 2–5 to 0–1 was considered significant symptom control.

Tumor markers, CGA and 24 h urine 5-HIAA, levels prior to dose escalation were documented and compared to the median of three subsequent tumor marker levels after dose escalation when available. Tumor marker values that were obtained prior to 3 months post dose escalation were excluded, as these values may reflect an effect of the previous dose. A reduction in 10% of the predose tumor marker value was chosen. This nonvalidated cut-point was chosen in order to differentiate between responsive and nonresponsive patients as fluctuations in these values of greater than 10% would be less likely to occur due to normal variability alone. The aggregate median levels of tumor markers before and after dose escalation was also calculated.

Radiological imaging reports of the tumor and metastasis were retrospectively reviewed. The size of the tumor prior to dose escalation was compared to imaging after dose escalation. Tumors were classified as “responsive” if tumor regression was observed, as “stable” if no new lesions or stable disease was documented, and “progressive” if a comment of disease progression was made in the radiology report.

## Results

### Patient characteristics

In the 6-year period between 2005 and 2011, 265 patients received a prescription for O-LAR, and 37 (14%) patients received at least one dose escalation of O-LAR beyond 30 mg. Characteristics of the patients who received greater than 30 mg IM per month of LAR are outlined in Table 1.

**Table 1:** Baseline characteristics of the cohort

	*N*	%
Total no. of patients	37	
Male	23	62.2
Female	14	37.8
Median age, *n* (range)	60 (30–87)	
Tumor origin		
Small bowel	18	48.6
Unknown	8	21.6
Pancreas	7	19
Large bowel	2	5.4
Lung	2	5.4
Metastasis		
Liver plus other organ	33	89.2
Liver only	19	51.4
Mesentery	8	21.6
Peritoneum	2	5.4
Bone	2	5.4
Brain	1	2.7
Orbit	1	2.7
No metastasis	1	2.7
Tumor type		
Carcinoid	31	83.8
Other neuroendocrine	6	16.2
Reason for dose escalation		
Diarrhea	16	29.1
Flushing	11	20
Progression	10	18.2
Increased tumor marker(s)	8	14.3
Prior treatment		
Surgery	19	34.5
Chemotherapy	5	9
MIBG	3	5.5
Chemoembolization	3	5.5
Doses used		
40 mg	36	97.3
50 mg	3	8.1
60 mg	16	43.2

MIBG, metaiodobenzylguanidine

The median age of diagnosis was 60 and males represented 62% of the population. The site of tumor origin was predominantly the small bowel (49%) and almost 90% of the patients experienced liver metastasis. All patients had metastatic disease. About half of the patients had prior surgery and a minority had other modalities of treatment prior to receiving dose-escalated O-LAR. The median time to first dose escalation beyond 30 mg O-LAR was 13 months (range of 1–66 months).

### Dose escalation events

A total of 55 dose escalation events were observed among the 37 eligible patients. A dose escalation event was defined as an increase in O-LAR dose followed by a period of observation to determine clinical, biochemical, and/or radiographic response. The cohort was followed every 3 months with median duration of follow-up of 2 years post dose escalation. Although in couple of patients the follow-up was only couple of months post dose escalation as their dose was escalated to higher doses of O-LAR, some patients were followed up to 7 years post dose escalation. The reasons for dose escalation included symptoms of diarrhea, flushing, bronchoconstriction, abdominal pain, as well as increased tumor marker measurements and tumor progression. Thirty-six of 37 patients received an initial dose escalation dose of 40 mg of octreotide LAR (97%). Sixteen (43%) patients received a maximal dose of 60 mg O-LAR IM every month. None of the patients received a dose higher than 60 mg due to guidelines in place limiting the maximal dose.

### Symptom response

**Figure 1:**
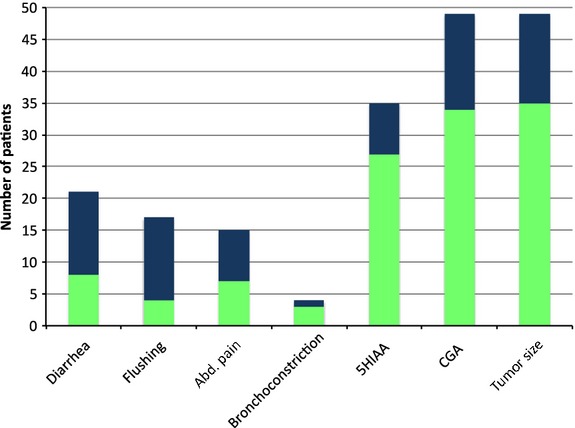
Characteristics of the patients who received greater than 30 mg IM per month of LAR. Number of patients with improvement in symptoms, tumor markers, and tumor burden. The vertical axis denotes the number of patients. The light green portion of the column indicates the proportion of patients with no improvement in symptoms/5-HIAA/CGA, while the dark blue portion denotes improvement. For “tumor size” the light green denotes tumor progression, while the dark blue denotes stable disease. IM, intramuscular; LAR, long-acting release; 5-HIAA, 5-hydoxyindoacetate; CGA, chromogranin A.

Of 13 patients with diarrhea, 8 (62%) reported a significant decrease in their symptoms after initial dose escalation. Five (63%) of the eight patients with diarrhea who received subsequent dose escalation of O-LAR to 60 mg reported significant decrease or even resolution of their symptoms. Ten (91%) of the 11 who had flushing reported a marked decrease in their symptoms upon initial dose escalation. Of five patients with flushing who were treated with further dose escalation to 60 mg, 2 (40%) had significant decrease in their symptoms. Symptoms of bronchoconstriction were improved in one (25%) of the four who had an O-LAR dose escalation and abdominal pain was improved in eight (53%) of the 15 patients (Table 2, Fig. 1).

**Table 2:** Number of patients with NETs who demonstrated symptom control, tumor markers, and radiological response after O-LAR dose escalation

	*N*	%
Post-DI symptom		
Diarrhea	13/21	62
Flushing	13/17	76
Abdominal pain	8/15	53
Bronchoconstriction	1/4	25
Post-DI tumor markers		
Post-DI 5-HIAA decrease	8/35	23
Post-DI CGA decrease	15/49	31
Post-DI tumor size		
Radiographical progression	35/49	71
Radiographical stable disease	14/49	29
Radiographical tumor regression	0/49	0

NETs, neuroendocrine tumors; O-LAR, octreotide long-acting release; 5-HIAA, 5-hydoxyindoacetate; CGA, chromogranin A.

### Tumor markers

**Figure 2:**
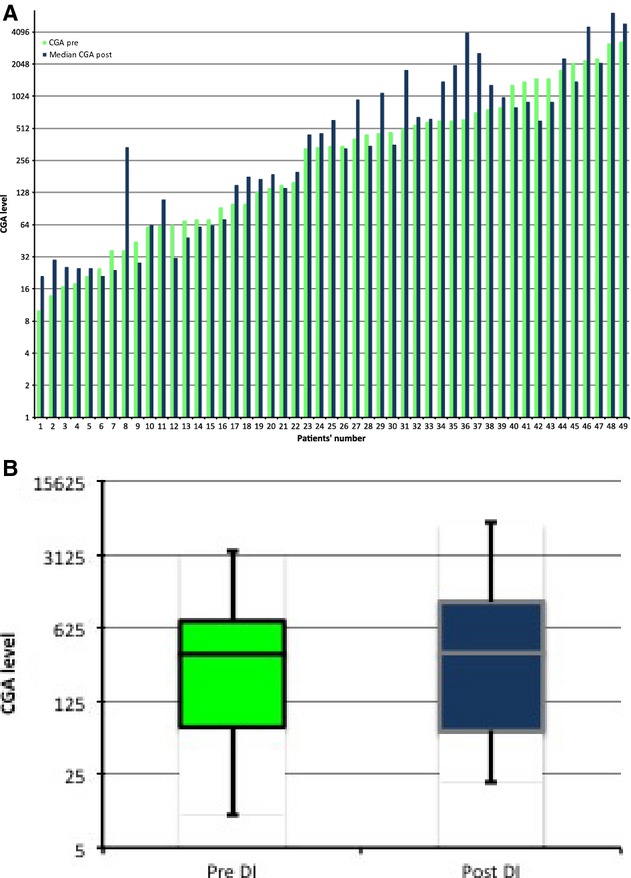
(A) Chromogranin A levels pre- and median post octreotide long-acting release dose escalation. (B) Box plot showing the median of aggregate values pre- and post dose escalation.

**Figure 3:**
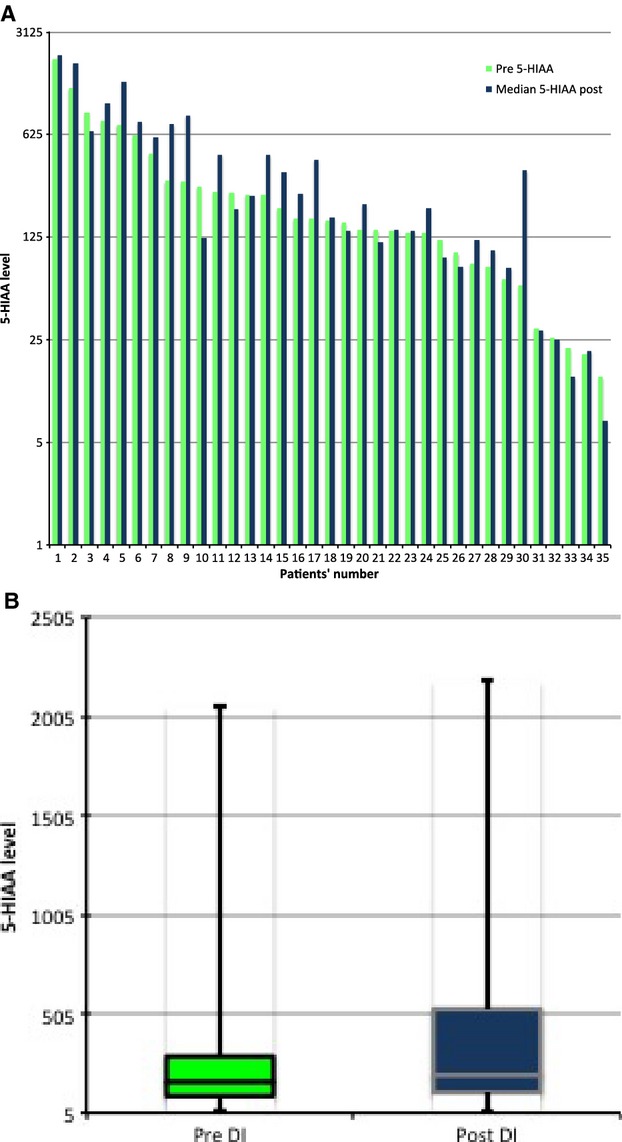
(A) 5-Hydoxyindoacetate levels pre- and median post octreotide long-acting release dose escalation. (B) Box plot showing the median of aggregate values pre- and post dose escalation.

Of the 49 dose escalation events where the CGA level was measured pre- and post dose escalation, 15 (31%) had a 10% decrease in their median level compared to pre-escalation levels. The remainder (69%) had either a stable or increased median levels. The median aggregate pre- and post dose escalation levels was 350 and 360, respectively. Thirty-five patients had a-HIAA level checked pre- and post dose escalation. Of these, eight (23%) had a decrease in their median 5-HIAA post dose escalation of at least 10% compared to the pre-escalation level. The median of aggregate pre- and post dose escalation levels was 162 and 197, respectively (Table 2, Figs.1-3).

### Tumor response

Of the patients who received 40 mg of O-LAR, 33 had radiological assessment pre- and post dose escalation. Of these, 27 (82%) had radiological progression and 6 (18%) had radiological stable disease. Of the patients who received 60 mg of O-LAR, 14 had radiological assessment pre- and post dose escalation. Among these, 6 (43%) had disease progression and 8 (57%) had radiological stable disease (Table 2, Fig. 1).

## Discussion

Octreotide is a somatostatin analog that is used to control the symptoms of diarrhea, flushing, bronchoconstriction, and abdominal pain in NET patients. We have conducted a retrospective chart review of patients who received a dose of O-LAR above 30 mg to better describe the British Columbian provincial experience of O-LAR dose escalation and its effect on symptom control, tumor markers, and radiological progression.

This study found that 14% of patients who initiated O-LAR therapy received dose escalations beyond 30 mg per month. Dose escalation to 40–60 mg of O-LAR controlled the symptoms of diarrhea and flushing in more than two-thirds of the patients whose symptoms were not controlled with a conventional dose of 30 mg. As provincial and national treatment guidelines recommend that O-LAR doses not be increased beyond 60 mg IM every month, the study was unable to assess higher doses. However, Strosberg et al. reported using doses as high as 160 mg q 4 weeks to control progressive symptoms 18.

Our study did not explore why some patients required higher doses of O-LAR for symptom relief. Potential reasons for requiring higher doses include increase in tumor burden, heterogeneity in somatostatin receptor expression, development of antibodies to octreotide, and development of a granulomatous reaction in the gluteus muscle leading to decreased absorption from the site. In clinical practice, resistance to somatostatin therapy is eventually observed in a subgroup of patients and generally requires other treatment strategies including tumor debulking or ablation, radiopeptide or systemic therapy 19. While surgical resection of the primary and metastatic lesions remains the mainstay of treatment, resection is not always possible and there is significant heterogeneity in NET prognosis and treatment strategies 20–23. Multiple therapeutic approaches have been developed for patients with inoperable disease including somatostatin therapy, chemotherapy, immunotherapy, peptide receptor radionuclide therapy, and ablative therapy mainly targeting the liver 24–26.

To date, this is the only study describing the tumor response and biomarker response to O-LAR dose escalation. CGA and urinary 5-HIAA are the most widely used tumor markers for NET. We measured the levels of these tumor markers both pre- and post dose escalation in this patient cohort, but found that CGA and 5-HIAA levels after dose escalation were variable, but increased in the majority (70% and 77%, respectively) of patients. 5-HIAA was decreased in about 23% of patients and CGA was decreased in 30% of patients initially. Importantly, our study showed that the response of tumor markers on O-LAR dose escalation was variable and is not a reliable indicator of symptom relief.

Despite their widespread use as tumor markers, both CGA and 5-HIAA levels are affected by multiple tumor-related and tumor-unrelated factors. Well-differentiated NETs, including carcinoid tumors, are associated with elevated blood concentrations of CGA, which may increase with larger tumor burden 27. CGA is a sensitive but less specific marker in these tumors 28. The levels of CGA secretion vary on a day-by-day basis in both healthy subjects and those with NETs. Elevated urinary levels of 5-HIAA are highly specific for serotonin-producing carcinoid tumors, especially those arising from the midgut, but they are not particularly sensitive. In one study, only 73% patients with metastatic carcinoid tumors had elevated levels of 5-HIAA 29. Measuring 5-HIAA level is considered a useful diagnostic test in patients with carcinoid syndrome, however, its use in measuring tumor response to therapy is not established and serial measurements of 24-h urine 5-HIAA also do not correlate with symptomatic benefit from various treatments 30.

In this study, radiological response to dose escalation varied in the cohort with tumor size progression in the majority (71%) and stable tumor size in around one-third (29%) of cases. There was no evidence of tumor regression due to dose escalation. The observation that 29% of tumors were stable after therapy may not necessarily relate to dose escalation, but is likely related to the slow nature of growth of NETs. Phase III studies have demonstrated the value of standard doses of octreotide and lanreotide in delaying progression and even inducing a tumor response in a small proportion of patients 12,31. However, it is not established that higher doses can serve as salvage therapy once progression has occurred.

The results of our study are consistent with previous reports suggesting that dose escalation of O-LAR can be used to successfully manage patients with refractory NET symptoms. Anthony et al. and Strosberg et al. reported that dose escalation improved symptoms of diarrhea and flushing 4,18. In addition, Anthony et al. also reported 50–55% stable disease with dose escalation 4. Their study also found that dose escalation also controlled abdominal pain in metastatic disease.

The main limitations of the study is the retrospective nature of the chart review as it depends on estimation of severity of symptoms based on whatever was described in the chart rather than a designed symptom control tool or questionnaire. Also the interval for measuring tumor markers varied between clinicians. Data for some patients were incomplete; some patients included in the study did not have their tumor marker levels measured post dose escalation and some had less than three readings.

In summary, patients with refractory symptoms due to NETs benefited from dose escalation of octreotide LAR; however, based on the results of this study, dose escalation is recommended for symptom relief only. Tumor markers levels varied in response to dose escalation, were not correlated with symptom relief, and are likely not an accurate indication of disease burden alone. Higher doses of O-LAR were not generally helpful in halting disease progression. A prospective study is warranted to further verify the findings of dose escalations.

## References

[b1] Modlin IM, Lye KD, Kidd M (2003). A 5-decade analysis of 13,715 carcinoid tumors. Cancer.

[b2] Yao JC, Hassan M, Phan A, Dagohoy C, Leary C, Mares JE (2008). One hundred years after “carcinoid”: epidemiology of and prognostic factors for neuroendocrine tumors in 35,825 cases in the United States. J. Clin. Oncol.

[b3] Modlin IM, Champaneria MC, Chan AK, Kidd M (2007). A three-decade analysis of 3,911 small intestinal neuroendocrine tumors: the rapid pace of no progress. Am. J. Gastroenterol.

[b4] Anthony L, Vinik AI (2011). Evaluating the characteristics and the management of patients with neuroendocrine tumors receiving octreotide LAR during a 6-year period. Pancreas.

[b5] Reubi JC, Schaer JC, Waser B, Mengod G (1994). Expression and localization of somatostatin receptor SSTR1, SSTR2, and SSTR3 messenger RNAs in primary human tumors using in situ hybridization. Cancer Res.

[b6] Ruszniewski P, Ducreux M, Chayvialle JA, Blumberg J, Cloarec D, Michel H (1996). Treatment of the carcinoid syndrome with the long acting somatostatin analogue lanreotide: a prospective study in 39 patients. Gut.

[b7] Davis Z, Moertel CG, McIlrath D (1973). The malignant cardinoid syndrome. Surg. Gynecol. Obstet.

[b8] Arnold R, Simon B, Wied M (2000). Treatment of neuroendocrine GEP tumours with somatostatin analogues: a review. Digestion.

[b9] Arnold R, Trautmann ME, Creutzfeldt W, Benning R, Benning M, Neuhaus C (1996). Somatostatin analogue octreotide and inhibition of tumour growth in metastatic endocrine gastroenteropancreatic tumours. Gut.

[b10] Saltz L, Trochanowski B, Buckley M, Heffernan B, Niedzwiecki D, Tao Y (1993). Octreotide as an antineoplastic agent in the treatment of functional and nonfunctional neuroendocrine tumors. Cancer.

[b11] Eriksson B, Renstrup J, Imam H, Oberg K (1997). High-dose treatment with lanreotide of patients with advanced neuroendocrine gastrointestinal tumors: clinical and biological effects. Ann. Oncol.

[b12] Rinke A, Muller HH, Schade-Brittinger C, Klose KJ, Barth P, Wied M (2009). Placebo-controlled, double-blind, prospective, randomized study on the effect of octreotide LAR in the control of tumor growth in patients with metastatic neuroendocrine midgut tumors: a report from the PROMID Study Group. J. Clin. Oncol.

[b13] Anthony L, Johnson D, Hande K, Shaff M, Winn S, Krozely M (1993). Somatostatin analogue phase I trials in neuroendocrine neoplasms. Acta Oncol.

[b14] Chadha MK, Lombardo J, Mashtare T, Wilding GE, Litwin A, Raczyk C (2009). High-dose octreotide acetate for management of gastroenteropancreatic neuroendocrine tumors. Anticancer Res.

[b15] Imam H, Eriksson B, Lukinius A, Janson ET, Lindgren PG, Wilander E (1997). Induction of apoptosis in neuroendocrine tumors of the digestive system during treatment with somatostatin analogs. Acta Oncol.

[b16] Welin SV, Janson ET, Sundin A, Stridsberg M, Lavenius E, Granberg D (2004). High-dose treatment with a long-acting somatostatin analogue in patients with advanced midgut carcinoid tumours. Eur. J. Endocrinol.

[b17] US Department of Health and Human Services, NIoH, National Cancer Institute (2009).

[b18] Strosberg JR, Benson AB, Huynh L, Duh MS, Goldman J, Sahai V (2014). Clinical benefits of above-standard dose of octreotide LAR in patients with neuroendocrine tumors for control of carcinoid syndrome symptoms: a multicenter retrospective chart review study. Oncologist.

[b19] Pape U-F, Perren A, Niederle B, Gross D, Gress T, Costa F (2012). ENETS consensus guidelines for the management of patients with neuroendocrine neoplasms from the jejuno-ileum and the appendix including goblet cell carcinomas. Neuroendocrinology.

[b20] Madeira I, Terris B, Voss M, Denys A, Sauvanet A, Flejou JF (1998). Prognostic factors in patients with endocrine tumours of the duodenopancreatic area. Gut.

[b21] Mignon M, Ruszniewski P, Haffar S, Rigaud D, Rene E, Bonfils S (1986). Current approach to the management of tumoral process in patients with gastrinoma. World J. Surg.

[b22] Godwin JD (1975). Carcinoid tumors. An analysis of 2,837 cases. Cancer.

[b23] Zeitels J, Naunheim K, Kaplan EL, Straus F (1982). Carcinoid tumors: a 37-year experience. Arch. Surg.

[b24] Kvols LK, Moertel CG, O'Connell MJ, Schutt AJ, Rubin J, Hahn RG (1986). Treatment of the malignant carcinoid syndrome. Evaluation of a long-acting somatostatin analogue. N. Engl. J. Med.

[b25] Oberg K, Norheim I, Lind E, Alm G, Lundqvist G, Wide L (1986). Treatment of malignant carcinoid tumors with human leukocyte interferon: long-term results. Cancer Treat. Rep.

[b26] Ruszniewski P, Rougier P, Roche A, Legmann P, Sibert A, Hochlaf S (1993). Hepatic arterial chemoembolization in patients with liver metastases of endocrine tumors. A prospective phase II study in 24 patients. Cancer.

[b27] Modlin IM, Gustafsson BI, Moss SF, Pavel M, Tsolakis AV, Kidd M (2010). Chromogranin A–biological function and clinical utility in neuro endocrine tumor disease. Ann. Surg. Oncol.

[b28] O'Toole D, Grossman A, Gross D, Delle Fave G, Barkmanova J, O'Connor J (2009). ENETS consensus guidelines for the standards of care in neuroendocrine tumors: biochemical markers. Neuroendocrinology.

[b29] Feldman JM, O'Dorisio TM (1986). Role of neuropeptides and serotonin in the diagnosis of carcinoid tumors. Am. J. Med.

[b30] Siperstein AE, Berber E (2001). Cryoablation, percutaneous alcohol injection, and radiofrequency ablation for treatment of neuroendocrine liver metastases. World J. Surg.

[b31] Caplin ME, Pavel M, Ćwikła JB, Phan AT, Raderer M, Sedláčková E (2014). Lanreotide in metastatic enteropancreatic neuroendocrine tumors. N. Engl. J. Med.

